# Long noncoding RNA ABHD11-AS1 functions as a competing endogenous RNA to regulate papillary thyroid cancer progression by miR-199a-5p/SLC1A5 axis

**DOI:** 10.1038/s41419-019-1850-4

**Published:** 2019-08-14

**Authors:** Xi Zhuang, Houchao Tong, Yu Ding, Luyao Wu, Jingsheng Cai, Yan Si, Hao Zhang, Meiping Shen

**Affiliations:** Department of General Surgery, The First Affiliated Hospital with Nanjing Medical University, Nanjing, Jiangsu PR China

**Keywords:** Oncogenes, Long non-coding RNAs

## Abstract

With the increasing incidence of papillary thyroid cancer (PTC), more attention has been paid to exploring the mechanism of PTC initiation and progression. In addition, ectopic expression of long noncoding RNAs (lncRNAs) is reported to play a pivotal role in multiple human cancers. Based on these findings, we examined lncRNA ABHD11 antisense RNA 1 (ABHD11-AS1) expression and its clinical significance, biological function and mechanism in PTC. First, we analyzed thyroid ABHD11-AS1 expression in The Cancer Genome Atlas (TCGA) and Gene Expression Omnibus (GEO) databases. Then, qRT-PCR was applied to detect the expression in paired PTC tissues and adjacent normal tissues, as well as in PTC cell lines (TPC-1 and K-1) and a normal thyroid follicular epithelium cell line (Nthy-ori3-1). In addition, we validated the relationship between ABHD11-AS1 expression and clinicopathological features by the Pearson X^2^ test. The oncogenic role of ABHD11-AS1 and its regulation of miR-199a-5p in PTC were examined by biological assays. Finally, bioinformatics analysis and mechanism assays were used to elucidate the underlying mechanism. We found that ABHD11-AS1 was remarkably overexpressed in PTC, and high expression was related to tumor size, lymph node metastasis, extrathyroidal extension and advanced TNM stage. Moreover, ABHD11-AS1 enhanced the abilities of cell proliferation, migration, and invasion, inhibited apoptosis in vitro, promoted tumorigenesis in vivo via sponging miR-199a-5p and then induced SLC1A5 activation. In addition, rescue assays were performed to confirm the ABHD11-AS1/miR-199a-5p/SLC1A5 axis. Taken together, the data show that ABHD11-AS1 acts as a competing endogenous RNA (ceRNA) to exert malignant properties in PTC through the miR-199a-5p/SLC1A5 axis. Therefore, our study may shed light on PTC diagnosis and therapies.

## Introduction

Thyroid cancer (TC) is the most common endocrine-related malignancy; it has a continuously increasing incidence and has attracted much attention from the public for several decades^1,2^. The histologic types of TC include differentiated (papillary and follicular thyroid carcinoma) and anaplastic thyroid carcinoma. Among these, PTC is the major type, accounting for <80% of cases^3^. Although fine-needle aspiration biopsy provides a fairly accurate preoperative diagnosis, 20–30% of cases are undetermined nodules with a risk of malignancy of 10–40%^4^. Moreover, nearly 59% of patients diagnosed with lymph node metastasis have a poor prognosis^5,6^. Although the main treatments, such as thyroidectomy, radioiodine therapy and thyroid-stimulating hormone (TSH) inhibition therapy, have resulted in great advancements, the prognosis of advanced patients is unsatisfactory^7^. Therefore, it is essential to explore a novel method for diagnosis and treatment.

LncRNAs are new biomarkers that have recently attracted much attention. They are transcripts of >200 nucleotides without protein-coding functions^8^. LncRNAs participate in various genetic processes, such as chromosome structure modulation, transcription, messenger RNA (mRNA) stability, mRNA availability and post-translational modification^9^. They regulate gene expression at the transcriptional, posttranscriptional and epigenetic levels^10^. For instance, lncRNA NEAT1 is upregulated in PTC tissues and cell lines and promotes cell proliferation, invasion, and migration and induces apoptosis by modulating miR-29-5p/KLK7 expression^11^. LncRNA CNALPTC1 is overexpressed in PTC, and its increased expression is associated with aggressive clinicopathological characteristics. In addition, CNALPTC1 promotes PTC progression via sponging members of the miR-30 family^12^. Hence, the specific mechanism of lncRNAs in PTC occurrence and progression requires further investigation.

ABHD11-AS1, which is located at 7q11.23, has been reported to be related to multiple cancers. For example, Lei et al. reported that the expression of ABHD11-AS1 was higher in colorectal cancer tissues and that silencing ABHD11-AS1 suppressed cell proliferation, migration, and invasion and induced apoptosis through the miR-133a/SOX4 axis^13^. It has also been reported that ABHD11-AS1 promotes cell proliferation, migration and EMT in pancreatic cancer by activating the PI3K-AKT pathway. High expression of ABHD11-AS1 has been correlated with distant metastasis, TNM stage, and tumor differentiation and predicts poor survival^14^. Moreover, the overexpression of ABHD11-AS1 in epithelial ovarian cancer was shown to promote tumorigenesis and progression by targeting Rhoc^15^.

In this study, we observed that the expression of ABHD11-AS1 was higher in PTC tissues and cells than in normal tissues and cells. By analyzing the relationship between ABHD11-AS1 and clinicopathological characteristics, we found that tumor size, lymph node metastasis, extrathyroidal extension and advanced TNM stage were related to the high expression of ABHD11-AS1. Loss-of-function assays indicated that ABHD11-AS1 promoted cell proliferation, migration, invasion, and EMT and reduced apoptosis in vitro and in vivo. Bioinformatics analysis together with biological experiments were performed to verify the underlying mechanism by which ABHD11-AS1 exerted its malignant properties. In short, ABHD11-AS1 acts as a ceRNA to sponge miR-199a-5p and then regulate SLC1A5 in PTC, suggesting that it can be a target for diagnosis and therapy.

## Materials and methods

### Patients and tissue samples

A total of eighty PTC tissues and paired adjacent normal tissues were obtained from patients who underwent surgery at Jiangsu Province Hospital, the First Affiliated Hospital of Nanjing Medical University (NMU). No patients received any local or systemic treatments before the operation. The adjacent non-cancerous tissues were collected >2 cm from the tumor margins on the same lobe or on the opposite lobe. All tissue samples were confirmed by two pathologists independently, frozen in liquid nitrogen immediately after surgical resection, and then stored at −80 °C until use for later experiments. The clinicopathological characteristics of the patients are listed in Table 1. Informed consent was obtained from all included patients, and the experiments were approved by the Research Ethics Committee of NMU.

**Table 1:** Correlation between ABHD11-AS1 expression and clinicopathological characteristics of thyroid cancer patients

Characteristics	Number	ABHD11-AS1 expression		*P* value
		High	Low	
Gender				
Male	21	11	10	0.799
Female	59	29	30	
Age (years)				
<45	41	20	21	0.823
≥45	39	20	19	
Multifocality				
Yes	16	8	8	1.000
No	64	32	32	
Tumor size (cm)				
≤2	66	29	37	0.019*
>2	14	11	3	
Lymph node metastasis				
Yes	39	24	15	0.044*
No	41	16	25	
Extral thyroidal extension				
Yes	15	12	3	0.010*
No	65	28	37	
TNM stage				
I + II	60	26	34	0.039*
III + IV	20	14	6	

*P ≤ 0.05 was considered significant

The bold values indicates that they have statistical significance

### Cell culture

Three PTC cell lines (K-1, TPC-1, and IHH-4) and a normal thyroid follicular epithelium cell line (Nthy-ori3-1) were obtained from the American Type Culture Collection (ATCC). Nthy-ori3-1 and K-1 cells were cultured in Roswell Park Memorial Institute (RPMI)−1640 medium (Gibco, USA) supplemented with 10% fetal bovine serum (WISENT, Canada). TPC-1 cells were cultured in DMEM high glucose (Gibco, USA) supplemented with 15% fetal bovine serum. A mixture (1:1) of RPMI 1640 and DMEM supplemented with 10% FBS was used to culture the IHH-4 cell line. A 1% antibiotic solution (100 U/ml penicillin and 100 mg/ml streptomycin) was added to all of the above culture media. All cells were cultured at 37 °C in a humidified atmosphere with 5% CO_2_.

### Total RNA extraction and quantitative real-time polymerase chain reaction (qRT-PCR) assays

Total RNA was extracted from PTC samples or cells using TRIzol reagent (Invitrogen, Carlsbad, CA, USA) according to the manufacturer’s instructions. Total RNA was reverse transcribed to complementary DNA (cDNA) using PrimeScript^TM^ RT Master Mix (TaKaRa, Japan). For miR-199a-5p, the Hairpin-it^TM^ miRNA qPCR Quantitation Kit (Genapharma, China) was applied to perform reverse transcription. Quantitative real-time PCR (qRT-PCR) was performed with AceQ qPCR SYBR Green Master Mix (Vazyme, China) and a 7500 Real-time PCR system (Applied Biosystems, Carlsbad, CA, USA). The relative expression levels of ABHD11-AS1 and SLC1A5 were normalized to GAPDH, while miR-199a-5p were normalized to U6. The specific primer sequences are listed in Table S1.

### Cell transfection

The ABHD11-AS1 (si-ANHD11-AS1) and SLC1A5 (si-SLC1A5) short interfering RNA (siRNA), miR-199a-5p inhibitor and corresponding negative controls (NCs) were synthesized by GenePharma (Shanghai, China). The short hairpin RNA designed against ABHD11-AS1 (sh-ABHD11-AS1) and its negative control (sh-NC) were inserted into lentiviral vectors constructed by GenePharma (Shanghai, China). All transfections were conducted using Lipofectamine 3000 (Invitrogen, Carlsbad, CA, USA) according to the manufacturer’s instructions, and 2 mg/ml puromycin was applied to select stably transfected cells. After transfection, the cells were harvested for the following experiments. The nucleotide sequences are listed in Table S1.

### Cell proliferation assay

Cell viability was measured by Cell Counting Kit-8 (DojinDO, Japan) assays every 24 h for four days following the manufacturer’s protocols. The transfected cells (1 × 10^3^ cells/well) were seeded into 96-well plates and cultured at 37 °C with 5% CO_2_. Ten microliters of CCK8 medium was added per well. After being cultured in an incubator for 2 h, the absorbance at 450 nm was detected using a spectrophotometer (Thermo Fisher, USA).

A total of 500 transfected cells were grown in 6-well plates and cultured in the appropriate medium for colony formation assays. Then, the colonies were fixed with methanol and stained with a 0.1% crystal violet solution (Beyotime, Nanjing, China) 14 days later. All visible colonies were counted manually.

### Ethynyldeoxyuridine (EdU) assay

Cell proliferation was also evaluated using an EdU assay kit (RiboBio, Guangzhou, China) following the manufacturer’s protocol. Cells were seeded in 96-well plates at a density of 5 × 10^3^ cells per well after transfection. Then, 50 µM EdU labeling medium was added to the cells, and the cells were cultured at 37 °C under 5% CO_2_ for 2 h. The cultured cells were fixed with 4% paraformaldehyde (pH = 7.4) for 30 min. Glycine was used to neutralize the paraformaldehyde and ensure the dyeing reaction. Next, 0.5% Triton X-100 was applied for permeabilization for 10 min at room temperature. After that, 1 × Apollo^®^ reaction cocktail (100 µl) was added to react with EdU for 30 min, and Hoechst 33342 (100 µl) was then added. Fluorescent microscopy (Nikon, Japan) was used to measure the percentage of EdU-positive cells in five random fields per well.

### Flow cytometric analysis

The apoptotic assay was conducted using an Annexin V-FITC/PI Apoptosis Detection Kit (KeyGEN BioTECH, Jiangsu, China). TPC-1 and K-1 cells were double stained with Annexin V-FITC and propidium iodide (PI) and analyzed with a flow cytometry (FACScan, BD Biosciences). The relative ratio of early and terminal apoptotic cells was detected to calculate the apoptotic rate. Transfected cells for the cell cycle assay were harvested, washed with PBS, and fixed with ethanol at −20 °C overnight. Then, the cells were stained with PI staining solution (MULTI SCIENCES, Zhejiang, China) and incubated for 30 min in the dark. The percentages of the cells in the G0/G1, S and G2/M phases were analyzed by flow cytometry.

### Cell migration and invasion assays

Cell migratory and invasive abilities were detected with Transwell chambers (Corning, USA) coated with or without Matrigel (BD Bioscience, USA). For the migration assay, transfected cancer cells (2 × 10^4^ cells/well) were suspended in 200 µl serum-free medium and seeded in the upper chambers. Then, 500 µl complete medium was added to the lower chambers as a chemoattractant. After being cultured in an incubator for 24 h, cells on the upper surface of the membrane were removed by cotton swabs, while cells that penetrated the lower surface were stained with crystal violet. An invasion assay was performed following the same steps above, except the upper surface of the membrane was coated with 0.1 µl Matrigel and incubated for 2 h. Five random fields were chosen for imaging, and we counted the cells on the bottom surface of the chamber under a microscope (Olympus, Japan).

### Wound healing assay

Transfected cells were plated in 6-well plates to conduct the wound healing assay. A scratch wound was created with a 200-µl sterile pipette tip, and the cells were washed with PBS several times. Then, the cells were cultured in serum-free medium. We imaged the wounds at the same position with a light microscope (Olympus, Japan) at 0 h and 24 h, and the area of the scratch was calculated.

### Tumor formation assay in vivo

All animal procedures were conducted in accordance with the Nanjing Medical University Institutional Animal Care and Use Committee. Ten four-week-old male BALB/c nude mice were purchased from the Animal Center of NMU and fed under specific pathogen-free conditions. TPC-1 cells stably transfected with sh-ABHD11-AS1 and sh-NC were suspended in phosphate-buffered saline (PBS) and injected subcutaneously into the same side armpit of each nude mouse (1 × 10^7^ cells/100 µl). The tumor volumes were measured by a Vernier caliper every three days and calculated with the following formula: tumor volume = (length × width^2^)/2. The nude mice were euthanized after 15 days, and the tumor volumes and weights were measured.

### Western blot analysis

Protein was extracted from transfected cells with RIPA lysis buffer (Beyotime, Shanghai, China) supplemented with phenylmethylsulfonyl fluoride (PMSF) (Roche, Shanghai, China). Proteins were separated by sodium dodecyl sulfate-polyacrylamide gel electrophoresis (SDS-PAGE) and transferred to polyvinylidene fluoride (PVDF) membranes (Millipore, Germany). After being blocked with 5% nonfat milk at room temperature for 2 h, the membranes were incubated at 4 °C overnight with specific primary antibodies against E-cadherin (1:1000), N-cadherin (1:1000), vimentin (1:500), CDK-2 (1:1000), CDK-4 (1:1000), cyclin D1 (1:1000), cyclin B1 (1:1000), Bcl-2 (1:1000), Bax (1:2000), Bcl-XL (1:1000), and SLC1A5 (1:2000) from Cell Signaling Technology (Beverly, MA, USA) and with GAPDH (1:1000) and β-actin (1:1000) from Beyotime (Shanghai, China). Then, the membranes were incubated with secondary antibodies (1:1000; Cell Signaling Technology) for 2 h at room temperature. Before and after incubation with secondary antibodies, the membranes were washed with TBST. An enhanced chemiluminescence (ECL) detection system (Thermo Scientific, USA) was applied to visualize the protein bands. GAPDH or β-actin was used as an internal control.

### Luciferase reporter assay

Complementary sequences containing a wild-type or mutant ABHD11-AS1 fragment and 3′ untranslated region (UTR) of SLC1A5 were synthesized and subcloned into the pGL3 luciferase reporter vector (Promega, USA). Cells seeded in 24-well plates were cotransfected with miR-199a-5p, miR-324-3p, miR-1913, miR-3907 or empty vector, as well as luciferase reporter plasmids containing wild-type or mutant ABHD11-AS1 and the 3′UTR of the SLC1A5 fragment, with Lipofectamine 3000 (Invitrogen, USA). Renilla luciferase activity was used as a reference control. Firefly and Renilla luciferase activities were analyzed by a dual-luciferase reporter assay system (Promega, USA) after 48 h of transfection according to the protocol.

### Bioinformatic analyses

TCGA data for thyroid cancer (http://cancergenome.nih.gov/) and the GSE66783 dataset for PTC (https://www.ncbi.nlm.nih.gov/geo/) were applied for screening differentially expressed lncRNAs. These data were also used for the preliminary analysis of ABHD11-AS1, miR-199a-5p and SLC1A5 expression in thyroid cancer tissues and normal tissues. Afterward, we used RegRNA 2.0 (http://regrna2.mbc.nctu.edu.tw/index.html) and lncRNASNP2 (http://bioinfo.life.hust.edu.cn/lncRNASNP/#!/) to predict the targeting relationship between lncRNA and miRNA. For the dual luciferase reporter assay, lncRNASNP2 was used to predict the binding site between ABHD11-AS1 and miR-199a-5p. Five online prediction tools, Targetscan, miRanda, miRDB, miRWalk and PITA (http://zmf.umm.uni-heidelberg.de/apps/zmf/mirwalk2/miRretsys-self.html), were used to determine the miRNA-mRNA relationships. In addition, the different prediction results were analyzed and used to draw Venn diagrams (http://bioinformatics.psb.ugent.be/webtools/Venn/). Moreover, the putative binding site between miR-199a-5p and SLC1A5 from miRWalk was applied for miRNA target validation assays.

### Statistical analysis

All statistical analyses were conducted using SPSS v19.0 and GraphPad Prism6, and the data are expressed as the mean ± standard deviation (SD). All experiments were performed independently at least three times. The significance of differences between groups was evaluated by Student’s *t* test, Pearson X^2^ test, Wilcoxon test and analysis of variance followed by Bonferroni post hoc test. Moreover, Spearman’s correlation analysis was conducted to analyze the correlations among ABHD11-AS1, miR-199a-5p and SLC1A5. *P* < 0.05 was considered statistically significant.

## Results

### ABHD11-AS1 is upregulated in papillary thyroid cancer tissues and cell lines

**Fig. 1:**
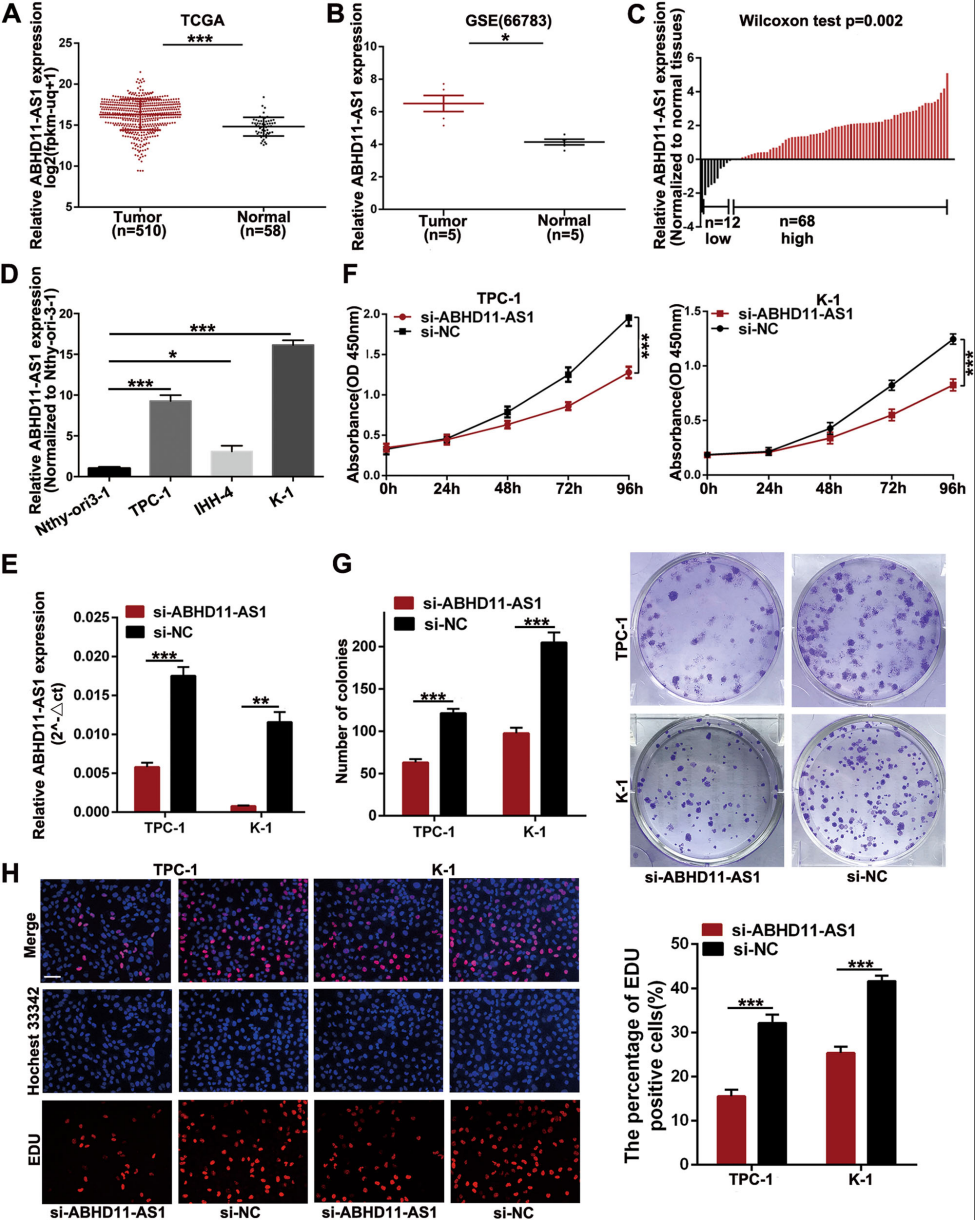
ABHD11-AS1 expression in thyroid cancer tissues and cell lines and its effects on PTC cells proliferation in vitro. **a**, **b** Relative expression of ABHD11-AS1 in thyroid cancer tissues and paired normal tissues were analyzed by TCGA and GEO (GSE66783) database. **c** The relative ABHD11-AS1 expression in PTC tissues and corresponding adjacent normal tissues (*n* = 80) were determined using qRT-PCR and normalized to GAPDH. The date was presented as the ∆C_t_ value. **d** qRT-PCR analysis of ABHD11-AS1 expression in normal thyroid follicular epithelial cells (Nthy-ori3-1) and three PTC cell lines. **e** The expression level of ABHD11-AS1 in TPC-1 and K-1 transfected with si-NC or si-ABHD11-AS1 were analyzed by qRT-PCR. **f** CCK-8 assays were conducted to detect the viability of thyroid cancer cells after transfection. **g**, **h** Colony formation assays and EDU staining assays were applied to detect the proliferation of si-ABHD11-AS1-transfected PTC cells (scale bar: 100um). The data is presented as mean ± SD (standard deviation) from three independent experiments. *P ≤ 0.05, **P ≤ 0.01, ***P ≤ 0.001

To evaluate the expression of thyroid cancer-related lncRNAs that may participate in thyroid cancer progression, we initially analyzed data from the TCGA and GEO (GSE66783) databases. The results indicated that compared with that in normal tissues, ABHD11-AS1 was significantly overexpressed in TC (Fig. 1a, b). To identify the expression level of ABHD11-AS1 in papillary thyroid cancer (PTC), 80 pairs of PTC and adjacent normal tissues were collected and used for qRT-PCR. Figure 1c reveals that compared with that in normal tissues, ABHD11-AS1 expression was relatively upregulated in 68 PTC tissues, whereas it was downregulated in 12 PTC tissues. We also examined ABHD11-AS1 expression in normal thyroid follicular epithelial cells (Nthy-ori3-1) and PTC cell lines (TPC-1, K-1 and IHH-4) and found that the expression of ABHD11-AS1 was significantly higher in PTC cells than in thyroid follicular epithelial cells (Fig. 1d). Among the three PTC cell lines, the expression in TPC-1 and K-1 cells was higher. Then, we assessed the correlations between the expression level and the clinicopathological features to explore the potential clinical significance of ABHD11-AS1 in PTC. The ABHD11-AS1 median expression value in PTC tissues was used as a cut-off value to divide 80 patients into the following two groups: a relatively high ABHD11-AS1 expression group and a relatively low ABHD11-AS1 expression group (*n* = 40 > median, *n* = 40 ≤ median). As shown in Table 1, high ABHD11-AS1 expression was significantly associated with tumor size, lymph node metastasis, extrathyroidal extension and advanced TNM stage. Based on these results, we deduced that ABHD11-AS1 might play a vital role in PTC.

### ABHD11-AS1 promotes papillary thyroid cancer cell proliferation

**Fig. 2:**
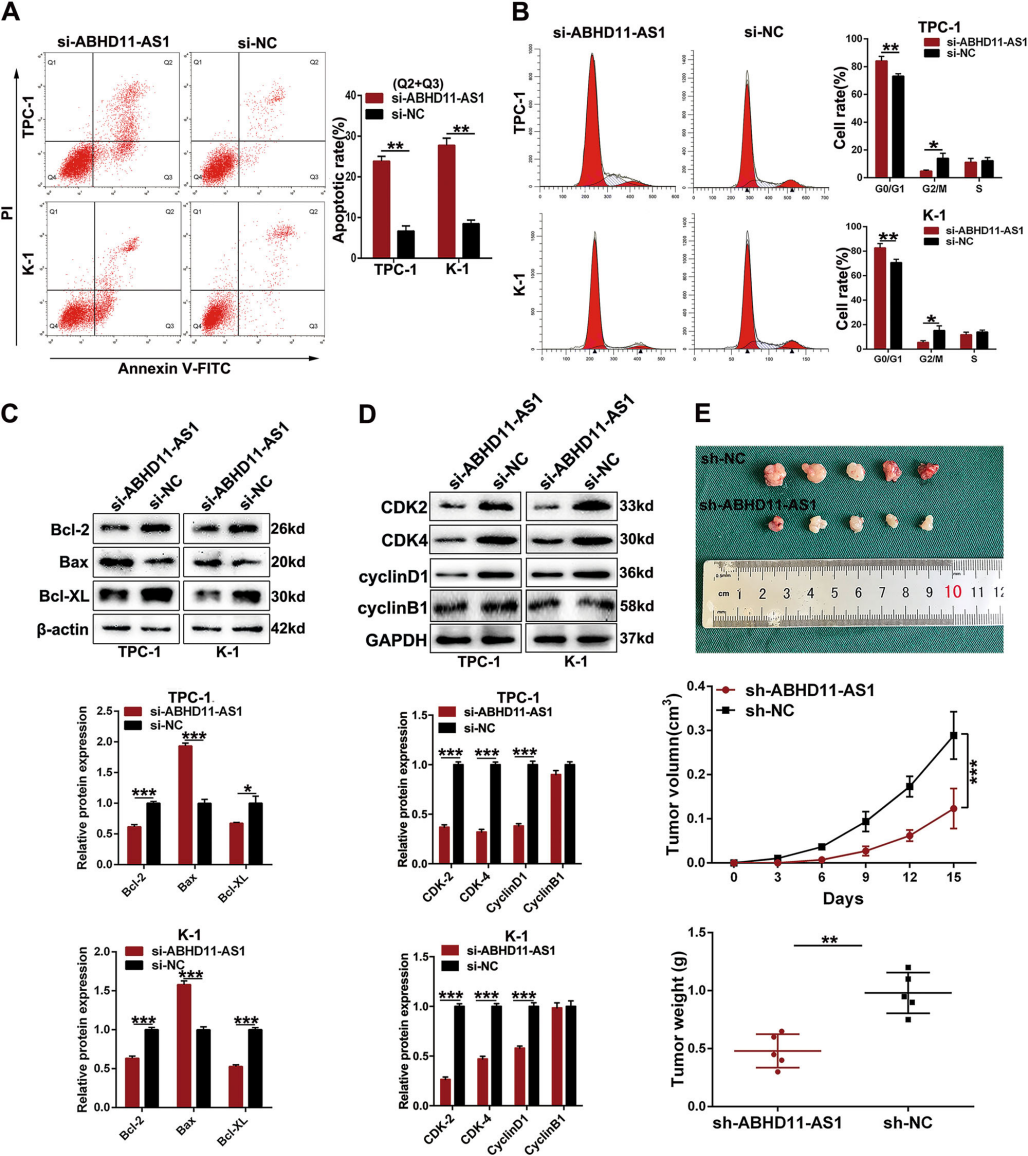
The effects of ABHD11-AS1 knockdown on PTC cells apoptosis and cell cycle in vitro and PTC tumorigenesis in vivo. **a** The apoptotic rates (LR + UR) of transfected cells were detected by flow cytometry(Q2 + Q3).LR, early apoptotic cells; UR, terminal apoptotic cells. **b** The increase or decrease in the proportion of transfected cell cycle was examined by flow cytometry. **c**, **d** Apoptosis-related protein and cell cycle-related proteins in PTC cells transfected with si-NC or si-ABHD11-AS1 were analyzed by western blot assays, GAPDH or β-actin protein was used as an internal control. **e** The effects of ABHD11-AS1 on tumor growth in vivo using cells transfected with sh-ABHD11-AS1 or sh-NC. The tumors from two groups’ nude mice were presented respectively. Tumor volumes was calculated after injection every three days and tumor growth curves were conducted. Tumor weights were measured after tumor removed. The data is presented as mean ± SD (standard deviation) from three independent experiments. *P ≤ 0.05, **P ≤ 0.01, ***P ≤ 0.001

To evaluate the effect of ABHD11-AS1 on PTC, we downregulated its expression by transfecting TPC-1 and K-1 cells, in which ABHD11-AS1 expression was relatively higher, with siRNA (si-ABHD11-AS1). After 48 h, we detected the silencing efficiency, and the results indicated that ABHD11-AS1 expression was remarkably lower in the si-ABHD11-AS1 groups than in the control groups of TPC-1 and K-1 cells (Fig. 1e). We conducted a CCK-8 assay to examine the roles of ABHD11-AS1 in cell proliferation. Cell growth curves showed that downregulating ABHD11-AS1 expression significantly inhibited cell growth compared with the negative control conditions (si-NC) (Fig. 1f). In agreement with the results of the CCK-8 assay, the colony formation assay revealed that clonogenic growth was obviously suppressed when ABHD11-AS1 was knocked down (Fig. 1g). EDU staining was also performed to confirm the proliferative ability of ABHD11-AS1, and the results showed that there were fewer EDU-positive cells in the si-ABHD11-AS1 group than in the si-NC group (Fig. 1h). Flow cytometric assays were performed to further elucidate the mechanism by which ABHD11-AS1 contributed to cell proliferation. As shown in Fig. 2a, TPC-1 and K-1 cells had higher apoptotic rates after transfection with si-ABHD11-AS1. Similarly, a cell cycle assay demonstrated that more cells were arrested in G0/G1 phase which resulted in fewer cells in G2/M phase compared with the si-NC group (Fig. 2b). In addition, the expression levels of apoptotic-related proteins (Bcl-2, BAX and Bcl-XL) and cell cycle-related proteins (CDK-2, CDK-4 and cyclin D1) were consistent with the respective cell apoptotic rate and cell cycle progression data. The change in the cyclin B1 protein expression level between the two groups had no statistical significance (Fig. 2c, d). All of the above data clarified that ABHD11-AS1 may promote the growth and survival of PTC cells.

### ABHD11-AS1 facilitates tumorigenesis in vivo

To investigate whether ABHD11-AS1 participated in tumor growth in vivo, TPC-1 cells transfected with shRNA (sh-ABHD11-AS1) and empty vector (sh-NC) were used to conduct a xenograft tumor formation assay. Transfected TPC-1 cells were injected subcutaneously into the same side flanks of ten nude mice, and all the nude mice formed tumors at the injection site. After 15 days of growth, we found remarkable decreases in tumor growth rates, sizes and weights in the sh-ABHD11-AS1 group compared to those in the sh-NC group (Fig. 2e). Herein, the data above revealed the oncogenic role of ABHD11-AS1 in vivo.

### ABHD11-AS1 promotes the migration and invasion of PTC cells

**Fig. 3:**
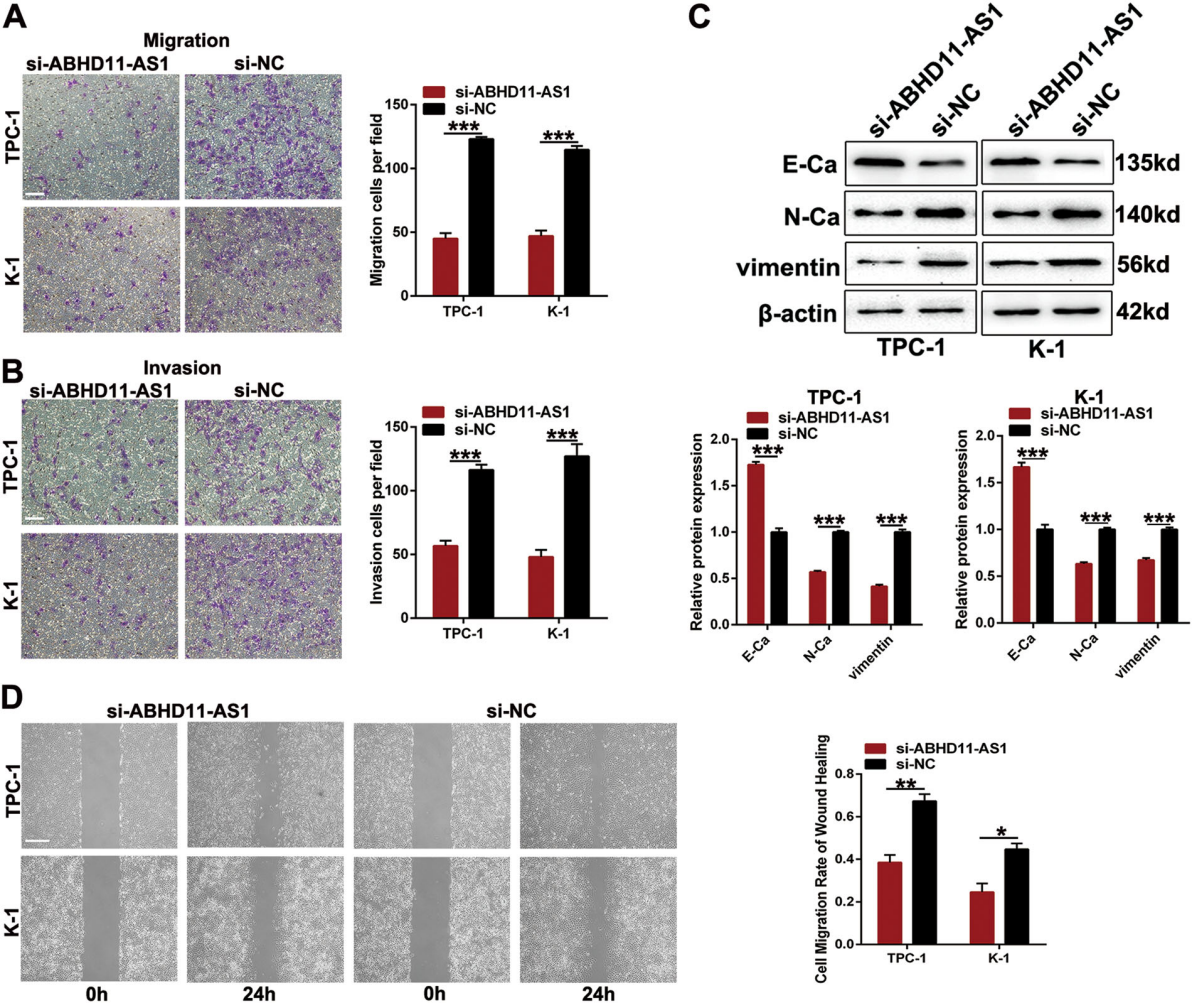
The effects of ABHD11-AS1 inhibition on thyroid cancer cells migration and invasion in vitro. **a**, **b** The capacities of migration and invasion was measured by transwell assays in PTC cells transfected with si-NC or si-ABHD11-AS1 (scale bar: 100 µm). **c** EMT-related protein was analyzed by western blot assays in transfected PTC cells. **d** Wound healing assays was also applied to examine cell migration ability (scale bar: 100 µm). The data is presented as mean ± SD (standard deviation) from three independent experiments. *P ≤ 0.05, **P ≤ 0.01, ***P ≤ 0.001

To detect other malignant properties of ABHD11-AS1 in PTC cells, we conducted transwell assays to examine its effects on migration and invasion. For the migration assay, the number of cells penetrating the lower surface in the si-ABHD11-AS1 group was less than that in the si-NC group (Fig. 3a). Figure 3b showed that compared with negative control cells, the number of invasive cells transfected with si-ABHD11-AS1 also decreased. Epithelial-mesenchymal transition (EMT) is closely connected with cell migration and invasion. Hence, we performed western blot assays to examine the relationship between ABHD11-AS1 and EMT-related proteins. As shown in Fig. 3c, ABHD11-AS1 knockdown decreased the expression of mesenchymal markers (N-cadherin and vimentin) and increased that of an epithelial marker (E-cadherin). Interestingly, we observed that the morphology of cells transfected with si-ABHD11-AS1 changed compared with si-NC group. Cells in si-ABHD11-AS1 group had epithelial phenotypes such as cobblestone-like shape and tight cell-cell adhesion which were consistent with EMT molecular markers (Fig. S2E). A wounding healing assay also indicated that cell migratory ability was suppressed after transfection with si-ABHD11-AS1 (Fig. 3d). In summary, these results indicated that migration and invasion behaviors might be promoted by ABHD11-AS1 in PTC cells.

### ABHD11-AS1 acts as a competing endogenous RNA (ceRNA) and sponges miR-199a-5p in papillary thyroid cancer

**Fig. 4:**
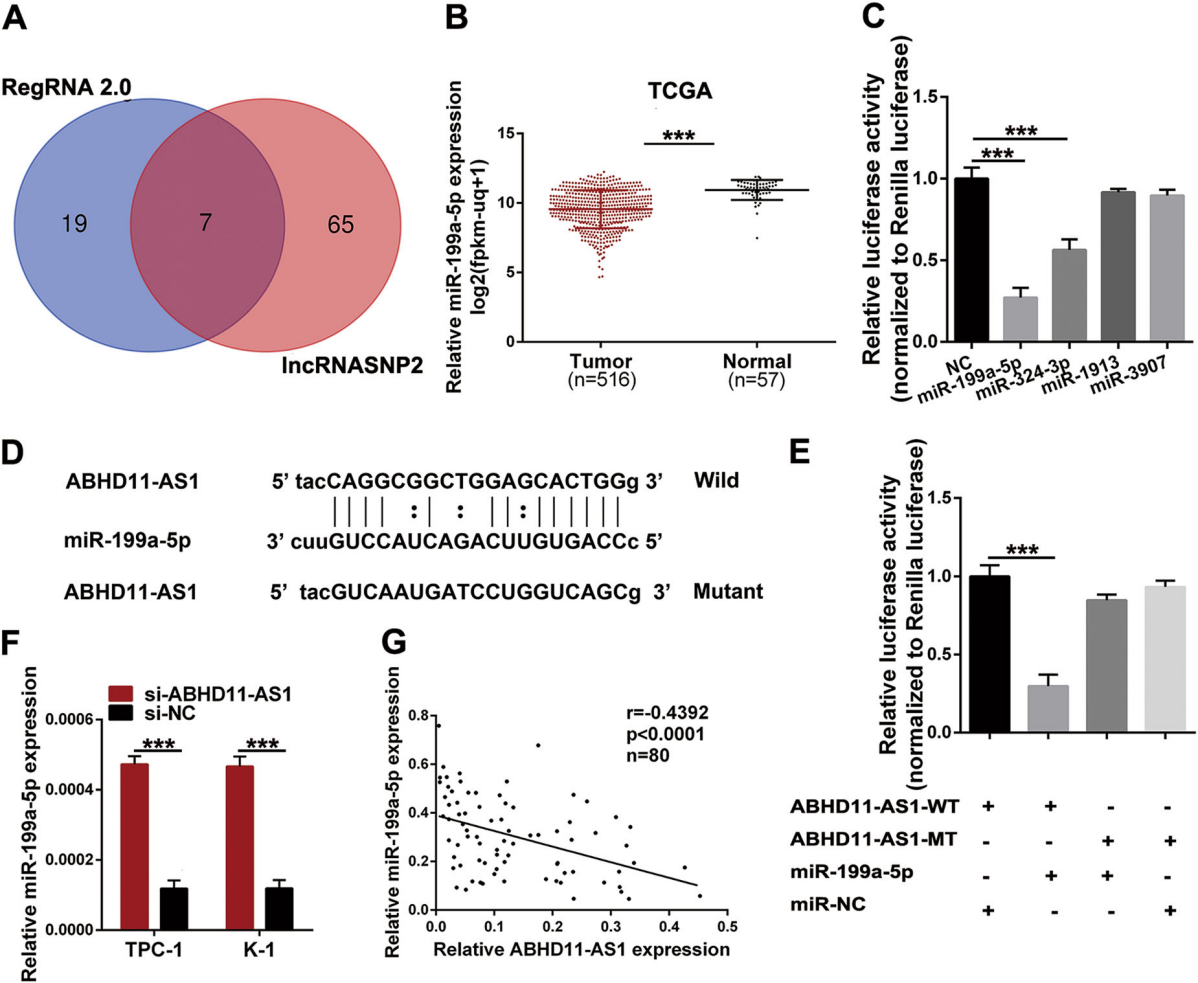
The interaction between ABHD11-AS1 and miR-199a-5p. **a** Two bioinformatics prediction tools (RegRNA 2.0 and lncRNASNP2) were used to screen 7 miRNAs which can bind with ABHD11-AS1. **b** The relative miR-199a-5p expression in cancer tissues compared with normal tissues was examined by TCGA database. **c** The luciferase reporter plasmids containing ABHD11-AS1 sequence was transfected with the 7 various miRNA-coding plasmids. **d** The predicted miR-199a-5p binding site in ABHD11-AS1 and its corresponding mutated sequences. **e** Dual luciferase reporter plasmid containing wild or mutant ABHD11-AS1 was cotransfected with miR-199a-5p as well as well as an empty plasmid vector. **f** The expression of miR-199a-5p in ABHD11-AS1 knockdown cells was analyzed by qRT-PCR. **g** The correlation analysis between ABHD11-AS1 and miR-199a-5p expression level. The data is presented as mean ± SD (standard deviation) from three independent experiments. *P ≤ 0.05, **P ≤ 0.01, ***P ≤ 0.001

To date, lncRNAs have been reported to participate in multiple molecular biological processes, such as RNA splicing, DNA replication and gene expression regulation. Moreover, a novel model called ceRNA has emerged as a popular research topic^16,17^. Emerging evidence has shown that ABHD11-AS1 acts as a ceRNA in PTC, yet lncRNA functioning as a ceRNA could sponge more than one miRNA to regulate multiple targets^18,19^. Therefore, the target prediction tools RegRNA 2.0 and lncRNASNP2 were applied to evaluate miRNAs that could have potential binding sites for ABHD11-AS1, and seven miRNAs were selected (Fig. 4a). According to the expression levels in thyroid cancer from the TCGA database, we found that among the 7 miRNAs selected, only miR-199a-5p was significantly lower in tumors than in normal tissues. Meanwhile, we selected four miRNAs (miR-199a-5p, miR-324-3p, miR-1913, and miR-3907) to conduct a dual luciferase reporter assay to further test the candidate miRNAs (Figs. 4b, S1A). The results indicated that the luciferase activity could be suppressed by miR-199a-5p and miR-324-3p, and the suppression ability of miR-199a-5p was stronger (Fig. 4c). To confirm the direct binding relationship between ABHD11-AS1 and miR-199a-5p, we constructed reporter plasmids containing the predicted miR-199a-5p wild-type or mutant binding sites (ABHD11-AS1-WT or ABHD11-AS1-MT), and these plasmids were cotransfected with miR-199a-5p or negative control miRNA. The putative binding sequences were obtained from lncRNASNP2 (Fig. 4d). As shown in Fig. 4e, the luciferase activity was significantly suppressed by miR-199a-5p in the presence of ABHD11-AS1-WT compared with that of the control, but this suppression did not exist in the presence of ABHD11-AS1-MT. Then, we conducted qRT-PCR to detect the expression of miR-199a-5p in tissues and cells with ABHD11-AS1 silencing. The results showed that miR-199a-5p was lower in PTC tissues than in matched normal tissues (Fig. S1B), and its expression level was significantly increased after ABHD11-AS1 knockdown (Fig. 4f). However, a miR-199a-5p inhibitor did not affect the expression of ABHD11-AS1 (Fig. S1C). According to Spearman’s correlation analysis, a negative relationship existed between ABHD11-AS1 and miR-199a-5p (Fig. 4g). Taken together, we propose that ABHD11-AS1 functions as a ceRNA by sponging miR-199a-5p.

### ABHD11-AS1 activity is directly and partially negatively modulated by miR-199a-5p

**Fig. 5:**
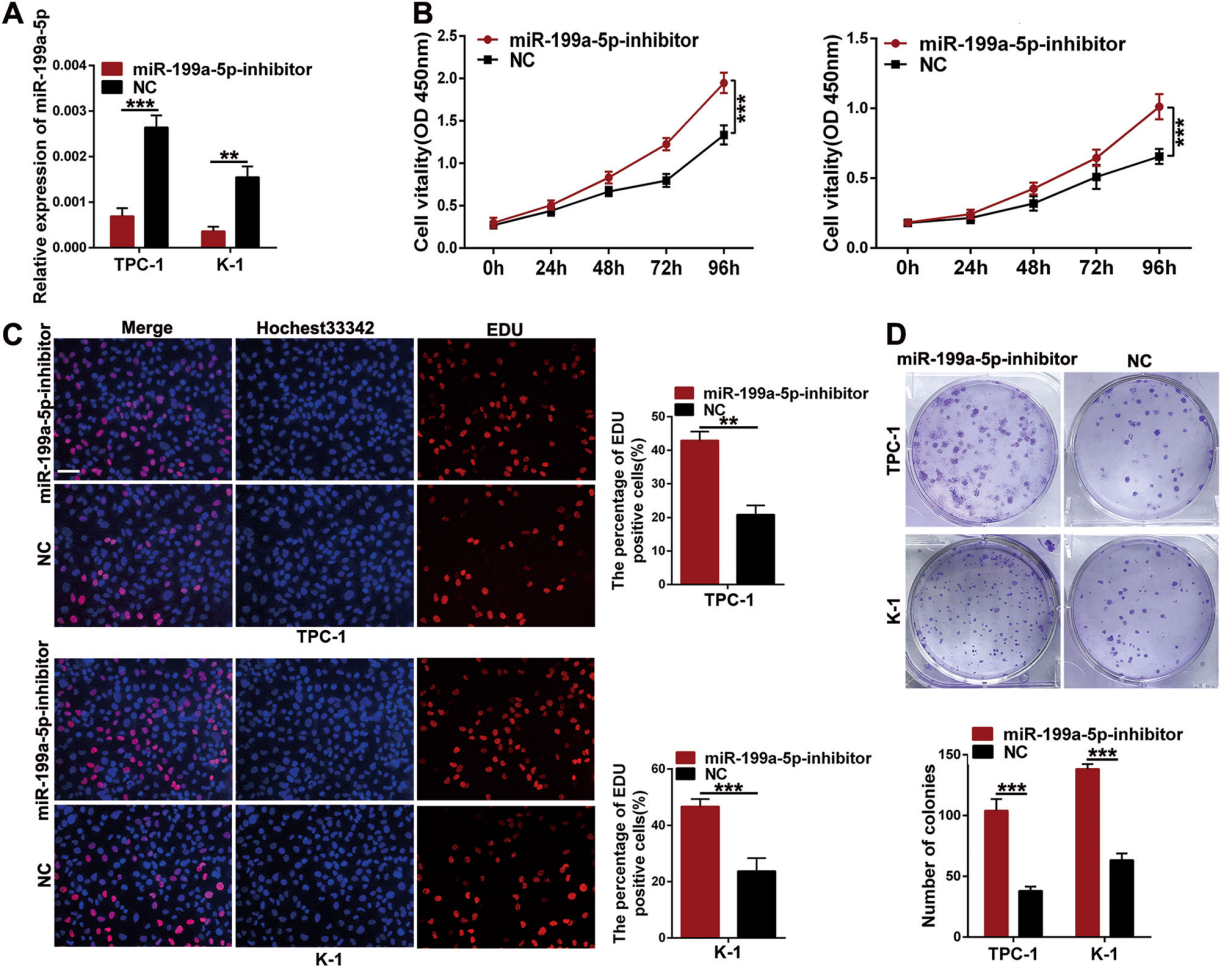
The effects of miR-199a-5p inhibited on PTC cells proliferation. **a** qRT-PCR was conducted to determine the knockdown efficiency in TPC-1 and K-1 cells after transfection of miR-199a-5p inhibitor and control miRNA (NC). **b** CCK-8 assays were performed to detect the viability of cells treated with miR-199a-5p inhibitor and NC. **c**, **d** Colony formation assays and EDU assays indicated that cell proliferation was promoted after being transfected with miR-199a-5p inhibitor compared with NC (scale bar: 100 um). The data is presented as mean ± SD (standard deviation) from three independent experiments. *P ≤ 0.05, **P ≤ 0.01, ***P ≤ 0.001

**Fig. 6:**
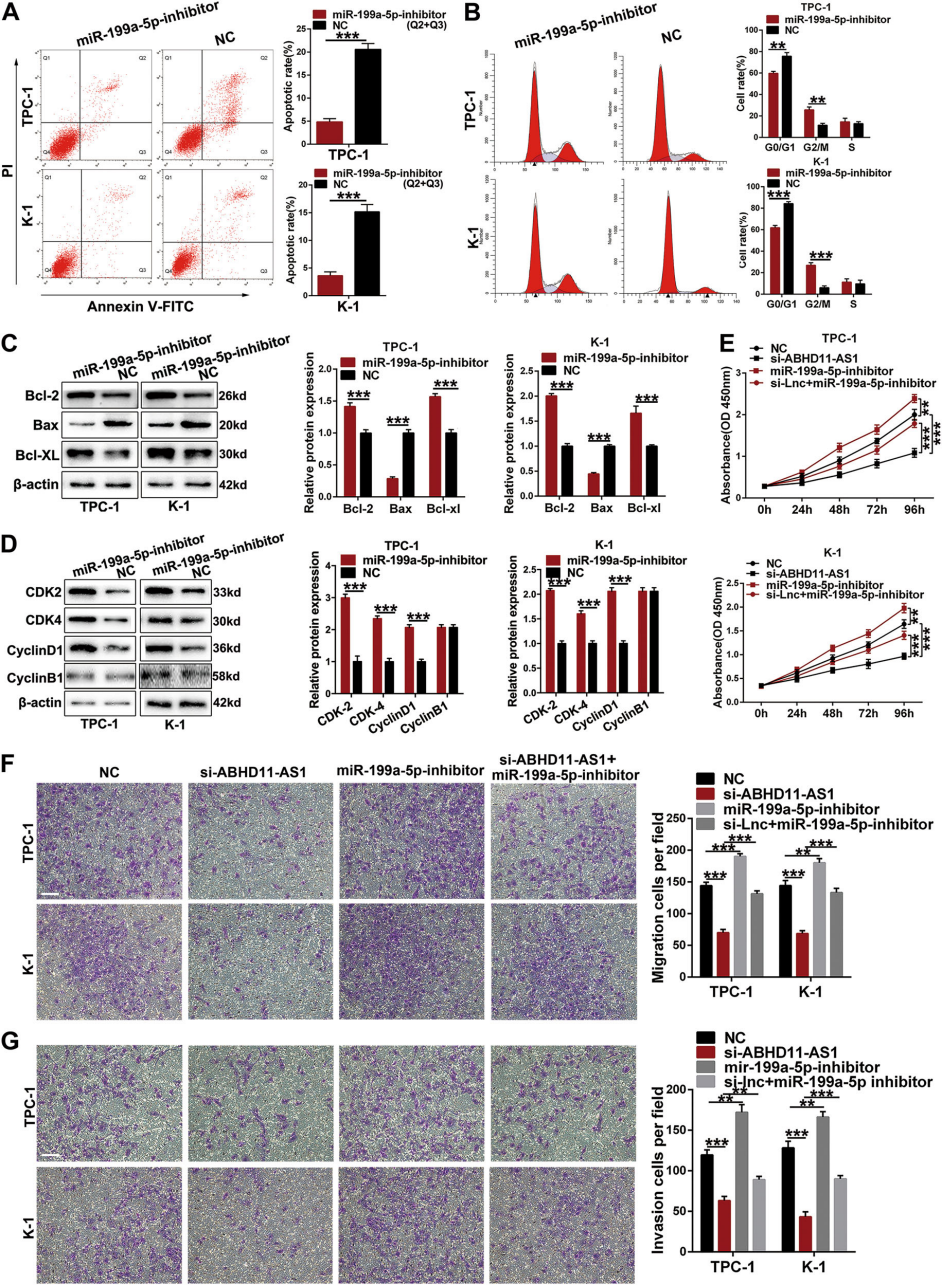
The effects of miR-199a-5p act on PTC cells progression and rescue ABHD11-AS1 properties. **a**, **b** Flow cytometric analysis was used to determine the apoptosis (LR + UR)(Q2 + Q3) and cell cycle in miR-199a-5p inhibitor or NC transfected cells respectively. LR, early apoptotic cells; UR, terminal apoptotic cells. **c**, **d** Western blot assays of apoptosis-related and cell cycle-related protein were conducted after miR-199a-5p inhibitor and NC transfection in TPC-1 and K-1 cells. **e**–**g** The proliferation, migration and invasion abilities of TPC-1 and K-1 cells after cotransfection with si-ABHD11-AS1, miR-199a-5p inhibitor and control siRNA were detected by CCK-8 assays and transwell assays (scale bar: 100 um). The data is presented as mean ± SD from three independent experiments. *P ≤ 0.05, **P ≤ 0.01, ***P ≤ 0.001. si-Lnc + miR-199a-5p-inhibitor: si-ABHD11-AS1 + miR-199a-5p-inhibitor

To determine whether the oncogenic effects of ABHD11-AS1 can be partially reversed by miR-199a-5p, we first examined the role of miR-199a-5p as a tumor suppressor in PTC. Ma et al. reported that miR-199a-5p could significantly suppress cell migration, invasion and EMT by binding to the 3′UTR of SNAI1 in PTC^20^; therefore, we performed CCK-8, EDU staining and colony formation assays to investigate the effects of miR-199a-5p on cell proliferation. TPC-1 and K-1 cells transfected with miR-199a-5p inhibitor and negative control (NC) were used for the following experiments, and the silencing efficiency is shown in Fig. 5a. The experimental results revealed that the miR-199a-5p inhibitor remarkably promoted cell proliferation and colony formation (Fig. 5b–d). Moreover, compared with that of the NC group, the apoptotic rate of cells was reduced, fewer cells were arrested in the G0/G1 phase, and more cells were in the G2/M phase in the miR-199a-5p inhibitor group according to flow cytometry analysis (Fig. 6a, b). Consistent with the results of cell apoptosis and cell cycle assays, silencing miR-199a-5p expression decreased the Bax protein level, whereas it increased the Bcl-2, Bcl-XL, CDK2, CDK4 and cyclin D1 protein levels. Moreover, compared with the NC group, the protein level of cyclin B1 did not change significantly after cells were transfected with the miR-199a-5p inhibitor (Fig. 6c, d). We conducted a rescue assay to validate whether the effects of ABHD11-AS1 on PTC cells could be modulated by the miR-199a-5p inhibitor. Then, si-ABHD11-AS1 and the miR-199a-5p inhibitor were cotransfected in TPC-1 and K-1 cells. CCK-8 and transwell assays showed that cell proliferation, migration and invasion were reduced by si-ABHD11-AS1 and reversed by the miR-199a-5p inhibitor (Fig. 6e–g). Thus, we concluded that ABHD11-AS1 exerts its function, at least partially, through sponging miR-199a-5p.

### SLC1A5, a target gene of miR-199a-5p, is regulated by ABHD11-AS1

**Fig. 7:**
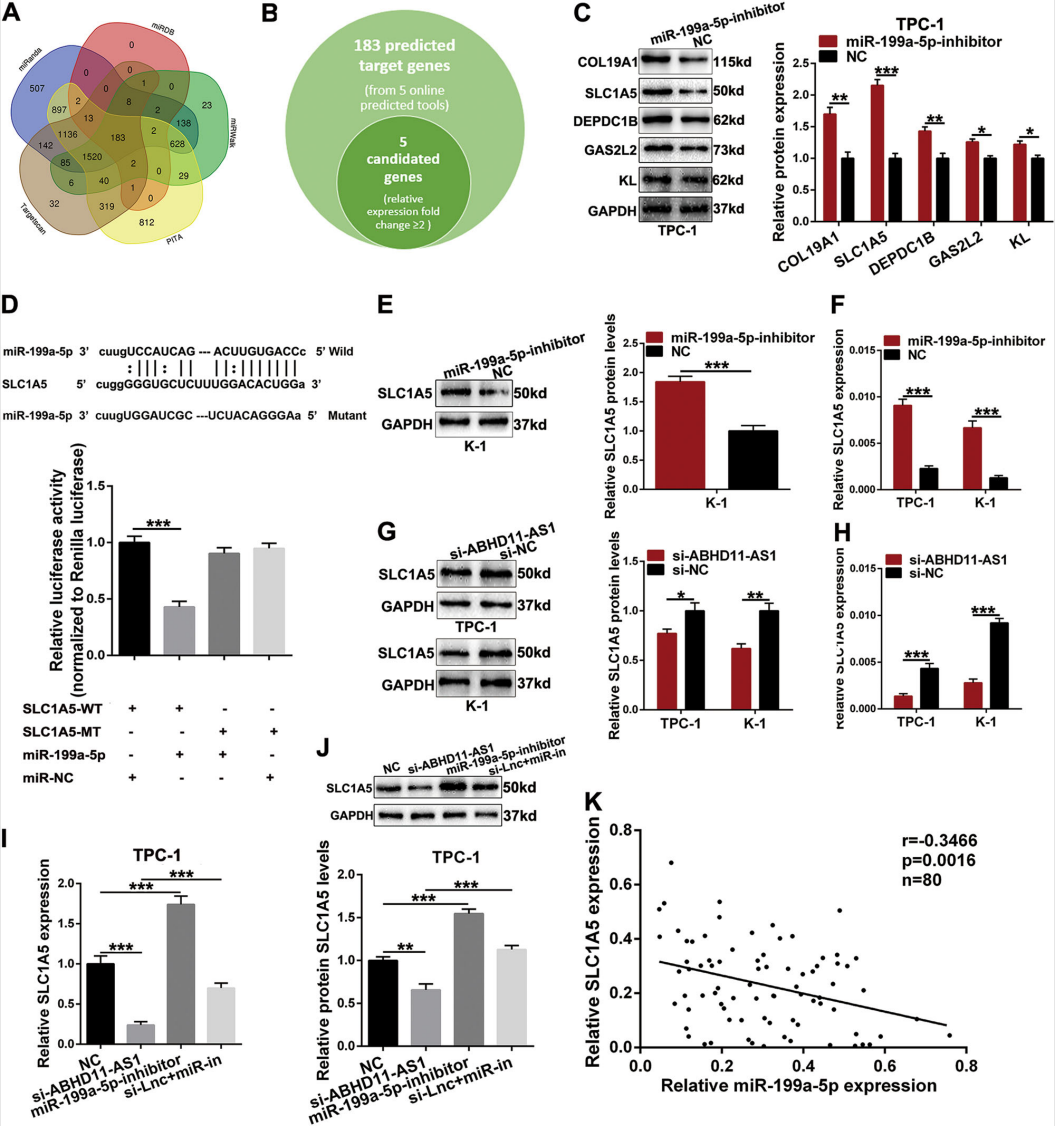
SLC1A5 is a target of miR-199a-5p and is regulated by ceRNA ABHD11-AS1. **a**, **b** 5 target genes that can bind with miR-199a-5p were selected out by five bioinformatics prediction softwares (Targetscan, miRanda, miRBD, miRWalk and PITA) and expression levels from TCGA databases. **c** The protein levels of 5 candidate target gene was detected after miR-199a-5p inhibitor and NC transfection in TPC-1 cells by western blot assays. **d** The miR-199a-5p putative binding site in the 3′-UTR of SLC1A5 (SLC1A5-WT) in line with its corresponding mutant transcripts (SLC1A5-MT). Luciferase reporter plasmids containing SLC1A5 3′-UTR (WT or MT) cotransfect with miR-199a-5p or miR-NC. **e**–**h** The relative expression of SLC1A5 were influenced by miR-199a-5p-inhibitor or si-ABHD11-AS1 relatively on mRNA and protein levels. **i**, **j** Rescue assays analyze SLC1A5 expression both on mRNA and protein levels after being cotransfected with si-ABHD11-AS1 and miR-199a-5p inhibitor in TPC-1. **k** The correlation analysis between miR-199a-5p and SLC1A5. The data is presented as mean ± SD (standard deviation) from three independent experiments. *P ≤ 0.05, **P ≤ 0.01, ***P ≤ 0.001. si-Lnc + miR-in: si-ABHD11-AS1 + miR-199a-5p-inhibitor

To further investigate the ceRNA network mechanism in papillary thyroid cancer (PTC), we used five online bioinformatics databases (Targetscan, miRanda, miRDB, miRWalk and PITA) to select target genes that can compete with ABHD11-AS1 to bind to miR-199a-5p. As presented in Fig. 7a, 183 target genes were predicted to have a binding site with miR-199a-5p. Among them, five target genes whose expression levels were markedly higher in thyroid cancer tissues than in normal tissues (*P* ≤ 0.05, fold change ≥ 2) were selected from the TCGA database (Fig. 7b). Next, western blot assays were conducted to examine the protein levels of the 5 potential target genes in TPC-1 cells transfected with the miR-199a-5p inhibitor, and we found that the SLC1A5 protein level was increased the most after conducting a multi-comparative analysis (Fig. 7c). Therefore, SLC1A5 was preliminarily chosen as a candidate target gene of miR-199a-5p for further analysis of PTC. To confirm the binding relationship between miR-199a-5p and SLC1A5, we performed dual luciferase reporter assays. Reporter plasmids containing the wild-type (WT) or mutant type (MT) SLC1A5 3′UTR (the latter contained a corresponding mutated sequence) were cotransfected with miR-199a-5p or negative control (miR-NC). Compared with miR-NC, the luciferase activity induced by miR-199a-5p contransfected the 3′UTR SLC1A5-WT was weakened, but this suppression did not exist in the plasmid with mutated binding site of 3′UTR SLC1A5 (Fig. 7d). Subsequently, we conducted qRT-PCR and western blot assays to determine whether SLC1A5 expression can be upregulated by miR-199a-5p inhibition. The results indicated that SLC1A5 expression was increased by miR-199a-5p inhibition at both the mRNA and protein levels compared with that in the negative control conditions in TPC-1 and K-1 cells (Fig. 7c, e, f). Therefore, we confirmed that SLC1A5 is a target gene of miR-199a-5p and can be regulated by miR-199a-5p.

The ceRNA mechanism posits that ABHD11-AS1 could be a sink for miR-199a-5p, thereby regulating SLC1A5 at the posttranscriptional level, so we explored whether the expression of SLC1A5 can be regulated by ABHD11-AS1. The data in Fig. 7g, h showed that SLC1A5 mRNA and protein levels were decreased by ABHD11-AS1 knockdown. We also detected the mRNA and protein expression levels of SLC1A5 in cells cotransfected with si-ABHD11-AS1 and miR-199a-5p inhibitor to examine the ABHD11-AS1/miR-199a-5p/SLC1A5 axis. As expected, the reduction in SLC1A5 expression driven by si-ABHD11-AS1 was dramatically reversed by the miR-199a-5p inhibitor (Figs. 7i, j, S1D–E). In parallel, we found a positive relationship between ABHD11-AS1 and SLC1A5, whereas a negative relationship was found between miR-199a-5p and SLC1A5 (Figs. 7k, S1F). Consequently, ABHD11-AS1 acts as a ceRNA to regulate SLC1A5 expression by sponging miR-199a-5p.

### SLC1A5 is overexpressed in papillary thyroid cancer and facilitates papillary thyroid cancer progression

**Fig. 8:**
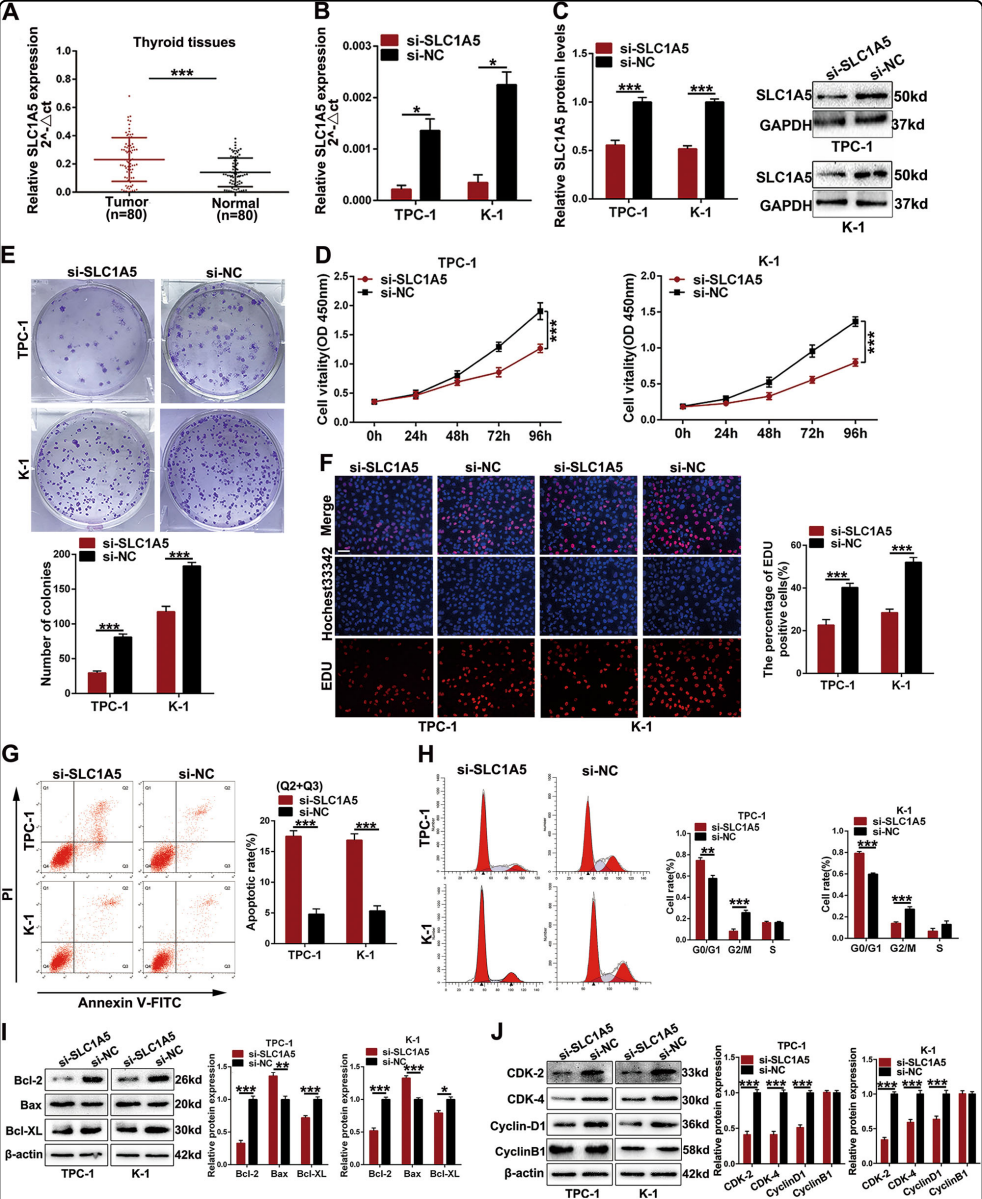
The malignant properties of SLC1A5 on thyroid cancer cells in vitro. **a** The relative SLC1A5 expression in thyroid cancer tissues and corresponding adjacent normal tissues (*n* = 80) were determined using qRT-PCR and normalized to GAPDH. **b**, **c** The knockdown efficiency in TPC-1 and K-1 cell was examined by qRT-PCR and western blot assays at mRNA and protein levels. **d**–**f** CCK-8, colony formation and EDU assays were used to determine the TPC-1 and K-1 cells growth after transfection si-SLC1A5 or si-NC (scale bar: 100 um). **g** The apoptotic rate of cells was examined by flow cytometry assays (Q2 + Q3). LR, early apoptotic cells, UR, terminal apoptotic cells. **h** Flow cytometry assay was also used to analyze cell cycle. **i**, **j** Western blot assays were used to determine the apoptotic-related and cell cycle-related protein levels in cells after transfection

**Fig. 9:**
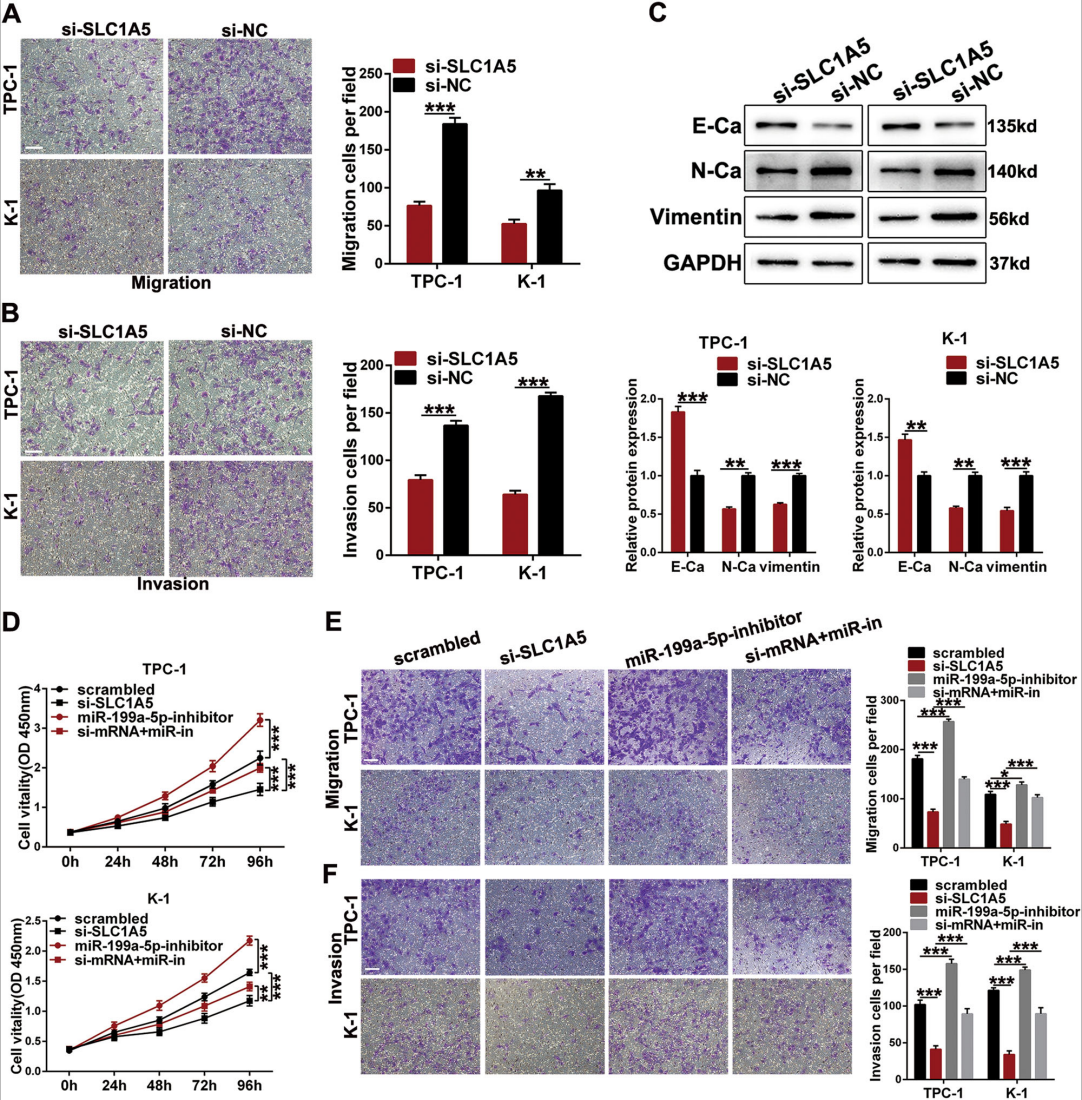
SLC1A5 facilitate PTC cells migration and invasion as well as promoted the PTC progression via ABHD11-AS1/miR-199a-5p/SLC1A5 axis. **a**, **b** The transwell assays were applied to examine the abilities of cell migration and invasion (scale bar: 100 µm). **c** EMT-related protein was analyzed by western blot assays in cells transfected with si-SLC1A5 or si-NC. **d** CCK-8 assay was used to analyze cell proliferation ability after cotransfection with si-NC, si-SLC1A5, or miR-199a-5p inhibitor. **e**, **f** Transwell assays was applied to examine cell migration and invasion properties in cells cotransfected with si-SLC1A5 and miR-199a-5p inhibitor (scale bar: 100 µm). The data is presented as mean ± SD (standard deviation) from three independent experiments. *P ≤ 0.05, **P ≤ 0.01, ***P ≤ 0.001. si-mRNA + miR-in: si-SLC1A5 + miR-199a-5p-inhibitor

The oncogenic effects of SLC1A5 on PTC have not been elucidated. Thus, we first detected SLC1A5 expression in thyroid cancer and normal tissues. As shown in Fig. S2A, the expression of SLC1A5 was upregulated in TC tissues according to TCGA and GSE (66783) database analyses. Similarly, SLC1A5 expression was significantly increased in 80 pairs of PTC tissues according to qRT-PCR results (Fig. 8a). Next, TPC-1 and K-1 cells transfected with SLC1A5 siRNA (si-SLC1A5) or negative control (si-NC) were used for subsequent loss-of-function experiments, and the efficiency was examined at the mRNA and protein levels (Fig. 8b, c). The CCK-8, colony formation and EDU staining assays showed that knockdown of SLC1A5 expression suppressed cell proliferation (Fig. 8d–f). In addition, we conducted flow cytometric analyses to detect the cell apoptotic rate and cell cycle. The apoptotic rate was obviously increased, and more cells were arrested in the G0/G1 phase after transfection with si-SLC1A5 (Fig. 8g, h). The results for apoptotic-related proteins (Bax, Bcl-2 and Bcl-XL) and cell cycle-related proteins (CDK2, CDK4, cyclin D1, and cyclin B1) were similar to those of the flow cytometric analyses (Fig. 8i, j). Moreover, we conducted transwell assays to investigate cell migration and invasion. The migratory and invasive properties introduced by silencing SLC1A5 were suppressed (Fig. 9a, b). A wound healing assay also indicated that the migratory ability was suppressed by silencing SLC1A5 (Fig. S2B). Western blot assays examined the expression of EMT-related protein in cells transfected with si-SLC1A5 or si-NC, and the results showed that an epithelial marker (E-cadherin) was increased, whereas mesenchymal markers (N-cadherin and vimentin) were decreased (Fig. 9c). Similarly, after transfected with si-SLC1A5, changes of PTC cells morphology were consistent with the expression of EMT-related protein (Fig. S2F).

In addition, the decreased mRNA and protein levels induced by si-SLC1A5 were effectively reversed by miR-199a-5p (Fig. S2C–D). CCK-8 and transwell assays were performed to further investigate whether the SLC1A5 activity in PTC could be influenced by miR-199a-5p. Experimental data showed that the inhibition of cell proliferation, migration and invasion induced by si-SLC1A5 was reversed by suppressing miR-199a-5p (Fig. 9d–f). These results indicate that SLC1A5 exerts malignant properties in PTC and can be partly modulated by miR-199a-5p.

## Discussion

Long noncoding RNAs (lncRNAs) play crucial roles in modulating gene expression. Moreover, they are involved in multiple biological processes, such as dosage compensation, genomic imprinting, and cell differentiation^21^. However, the aberrant expression of lncRNAs may result in the uncontrolled growth of abnormal cells and finally lead to tumorigenesis and progression^22^. Increasing studies have begun to focus on the effects of lncRNAs such as H19, HOXA-AS2, and SNHG15 in PTC^23–25^. Although it has been shown that ABHD11-AS1 acts as an oncogene in PTC, the regulatory mechanism has not been completely elucidated^19^. Here, we further investigated the malignant properties of ABHD11-AS1 in PTC and the underlying mechanism. Consistent with a previous study, we found that ABHD11-AS1 was significantly higher in 80 PTC tissues and cell lines than in paired normal tissues and normal thyroid follicular epithelial cells. High expression levels of ABHD11-AS1 significantly correlated with tumor size, lymph node metastasis, extrathyroidal extension and advanced TNM stage. Next, we conducted loss-of-function assays in two PTC cell lines. The experimental results showed that ABHD11-AS1 depletion suppressed cell proliferation, migration, invasion and EMT in vitro and tumor formation in vivo, whereas it promoted cell apoptosis. Collectively, the data indicate that ABHD11-AS1 plays an essential role in PTC progression.

LncRNAs exert oncogenic functions in cancers via a sophisticated mechanism; the ceRNA regulatory mechanism by which lncRNAs can sponge miRNAs and then release the miRNA target gene to facilitate tumor progression is predominant^9,26^. Moreover, several studies have confirmed that ABHD11-AS1 functions as a ceRNA to exert its regulatory function in multiple cancers. A previous study observed that ABHD11-AS1 acted as a ceRNA to promote PTC progression via the miR-1301-3p/STAT3 axis^19^. Nevertheless, as a ceRNA, ABHD11-AS1 can sponge more than one miRNA to exert its regulatory function. Therefore, we analyzed bioinformatics databases and found seven miRNAs that could contain ABHD11-AS1 binding sites. Based on the predicted expression level from TCGA and the results of the dual luciferase reporter assay, miR-199a-5p was selected as a candidate that ABHD11-AS1 can bind with. In addition, a reverse relationship existed between ABHD11-AS1 and miR-199a-5p based on Spearman’s correlation analysis. The tumor suppressor role of miR-199a-5p has been confirmed in many studies. For example, miR-199a-5p inhibited ovarian cancer cell proliferation and invasion by targeting NF-κB1^27^. Proliferation and metastasis abilities can be suppressed by miR-199a-5p in colorectal cancer via ROCK1 targeting^28^. Furthermore, Ma et al. showed that miR-199a-5p inhibited cell migration, invasion and EMT by targeting SNAI1 in PTC^20^. Therefore, we examined the effects of miR-199a-5p on cell proliferation. Biological assays indicated that miR-199a-5p attenuated cell proliferation, induced apoptosis and arrested cells in the G0/G1 phase in PTC. In addition, a rescue assay indicated that cell proliferation, migration and invasion were suppressed by silencing ABHD11-AS1, and these effects were counteracted by a miR-199a-5p inhibitor. Taken together, ABHD11-AS1 could sponge miR-199a-5p and block its tumor suppressor function.

In the ceRNA regulatory network, miRNAs ultimately modulate target gene activity via recognizing miRNA response elements (MREs) at the posttranscriptional level^29^. Subsequently, we devoted great effort to identifying target genes. In total, five online prediction tools were initially applied to seek miR-199a-5p targets. Then, we chose five of the 183 predicted target genes whose expression was remarkably upregulated in TC (*P* ≤ 0.05, fold change ≥ 2) to perform western blot assays. SLC1A5 was ultimately selected as a target gene of miR-199a-5p. To further confirm the direct relationship between miR-199a-5p and SLC1A5, we used a dual luciferase reporter assay. Moreover, the expression of SLC1A5 could be directly regulated by miR-199a-5p and indirectly regulated by ABHD11-AS1 at both the mRNA and protein levels. SLC1A5, also called ASCT2, is a Na^+^-dependent neutral amino acid transporter that is a member of the solute carrier family 1^30^. This gene mainly transports glutamine, which is essential for the growth of tumors that have increased metabolic demands^31^. Therefore, SLC1A5 functions as an oncogene in multiple human cancers. For instance, targeting SLC1A5 inhibited cell proliferation in melanoma, prostate cancer, triple-negative basal-like breast cancer and gastric cancer^32–35^. Esophageal cancer growth was suppressed by SLC1A5 silencing via cell cycle arrest and apoptosis^36^. SLC1A5 is also related to non-small cell lung cancer prognosis^37^. In thyroid cancer, SLC1A5 is reported to be expressed in only tumor cells and related to the BRAF V600E mutation, which is associated with extrathyroidal extension, advanced TNM stage, lymph node metastasis, multifocality and recurrence^38^. In this study, we detected the expression level of SLC1A5 in PTC. The results showed that SLC1A5 expression is higher in PTC than in normal tissues. Next, loss-of-function assays indicated that knockdown of SLC1A5 inhibited cell proliferation, migration, invasion and EMT, whereas it induced cell apoptosis. Furthermore, Spearman’s correlation analysis indicated that there was a positive correlation between ABHD11-AS1 and SLC1A5; nevertheless, there was a reverse relationship between ABHD11-AS1 and miR-199a-5p.

To validate the role of the ABHD11-AS1/miR-199a-5p/SLC1A5 axis in PTC, we performed rescue assays. The downregulated expression of SLC1A5 induced by silencing ABHD11-AS1 was abolished by a miR-199a-5p inhibitor. In parallel, the oncogenic effects inhibited by si-SLC1A5 were also reversed by the miR-199a-5p inhibitor.

In sum, we examined the expression of ABHD11-AS1 and its malignant properties in PTC. In addition, based on previous studies, we explored a novel mechanism in which ABHD11-AS1 exerted oncogenic functions via the ABHD11-AS1/miR-199a-5p/SLC1A5 axis. Therefore, this study further elucidated the ceRNA regulatory network in PTC and might provide a novel diagnostic and therapeutic target for PTC.

## Supplementary information

Figure S1

Figure S2

Table S1

Supplementary figure legends
