# Supplementary figures and images for: Long noncoding RNA ABHD11-AS1 functions as a competing endogenous RNA to regulate papillary thyroid cancer progression by miR-199a-5p/SLC1A5 axis
Source: Cell Death Dis. 2019 Aug 14;10(8):620. doi: 10.1038/s41419-019-1850-4 (PMC6692390; doi:10.1038/s41419-019-1850-4)

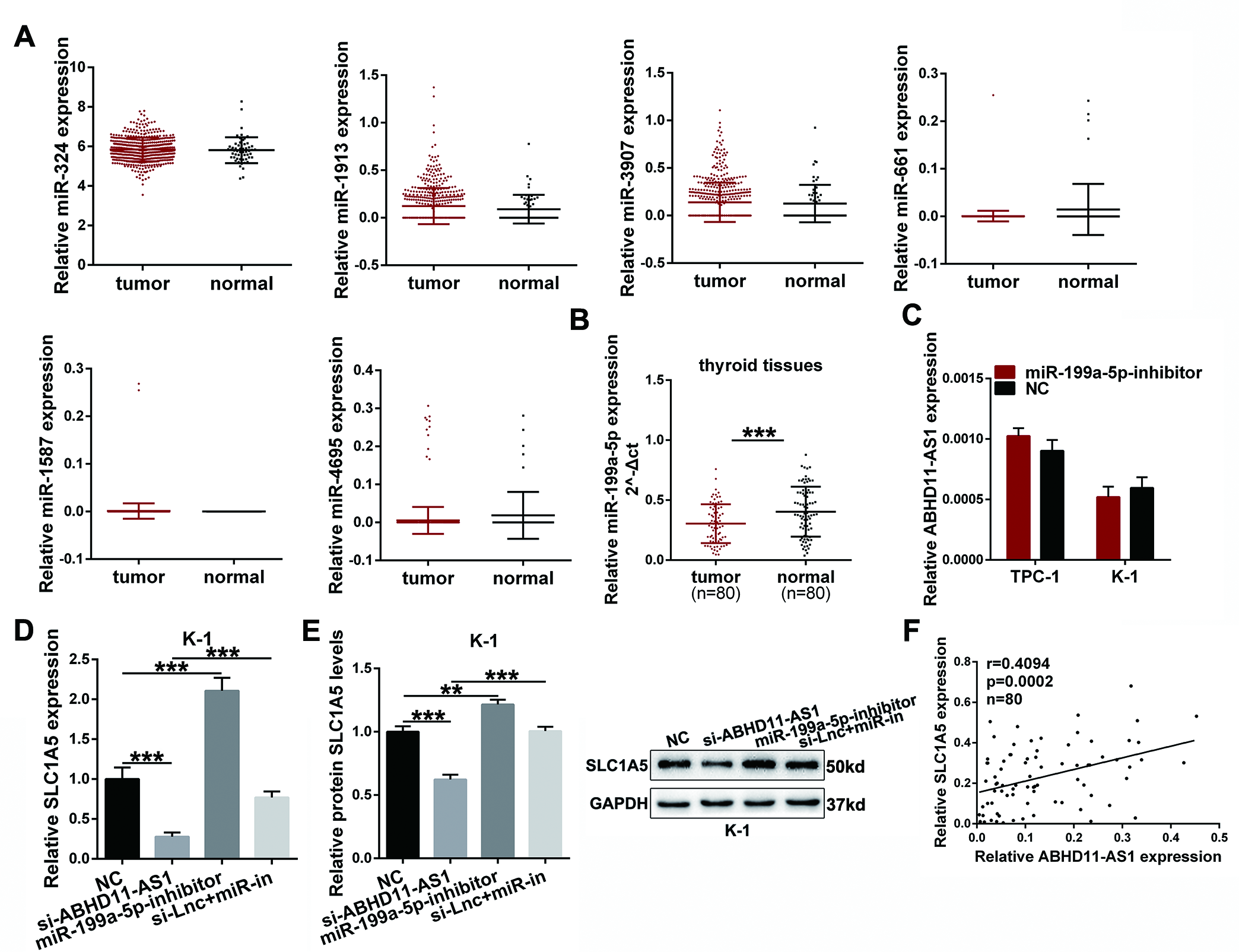

Supplement: Supplementary file 1 — Figure S1 [file 41419_2019_1850_MOESM1_ESM.tif]

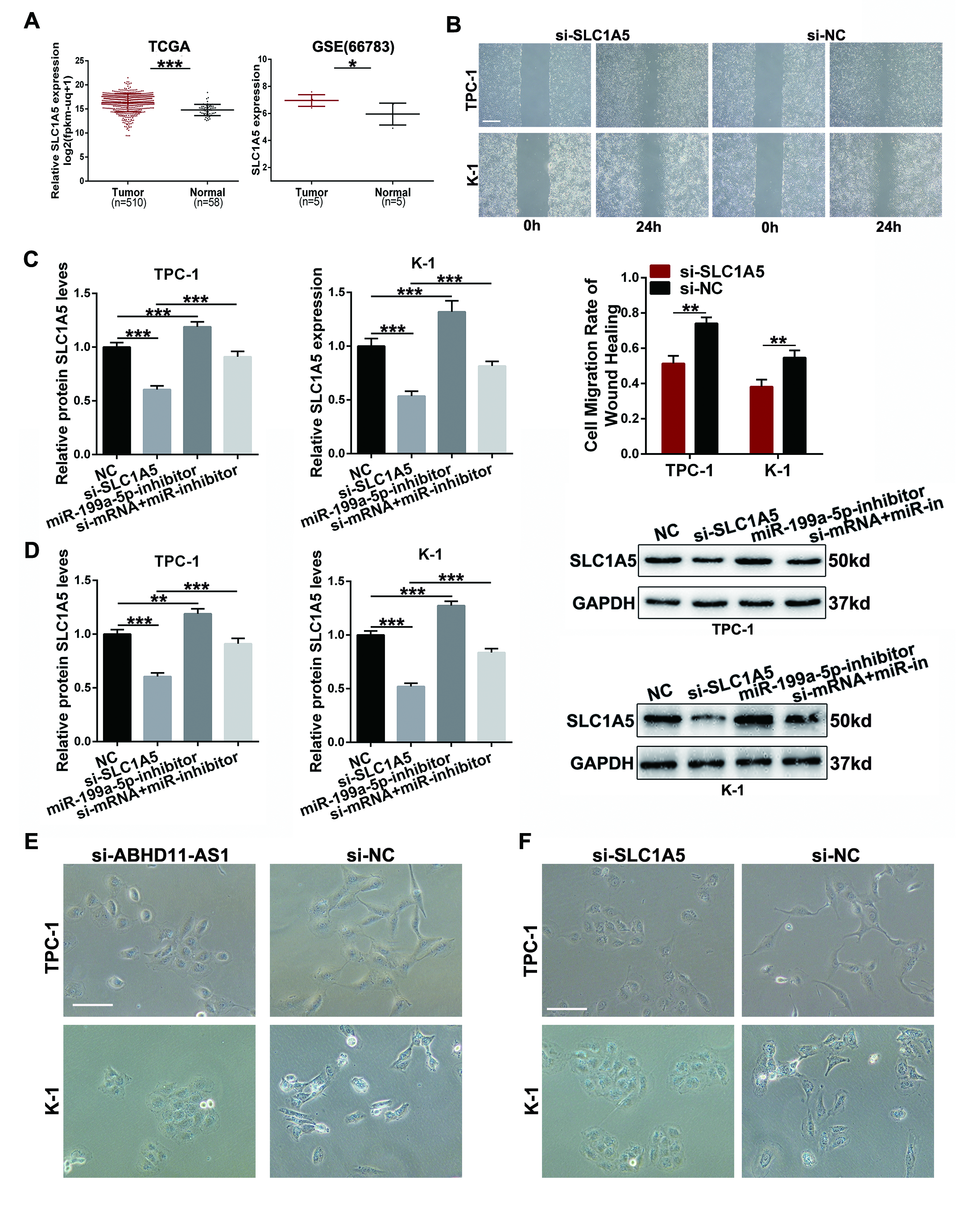

Supplement: Supplementary file 2 — Figure S2 [file 41419_2019_1850_MOESM2_ESM.tif]
