# Supplementary material for: Long noncoding RNA ABHD11-AS1 functions as a competing endogenous RNA to regulate papillary thyroid cancer progression by miR-199a-5p/SLC1A5 axis
Source: Cell Death Dis. 2019 Aug 14;10(8):620. doi: 10.1038/s41419-019-1850-4 (PMC6692390; doi:10.1038/s41419-019-1850-4)
Supplement: Supplementary file 4 — Supplementary figure legends [file 41419_2019_1850_MOESM4_ESM.docx]

**Supplementary Figure legend**

**Figure S1**

**A,** The remaining prediceted miRNAs expression from TCGA database. **B,** The expression of miR-199a-5p in 80 pairs thyroid tissues. **C,** qRT-PCR analysis of ABHD11-AS1 expression in cells transfected with miR-199a-5p-inhibitor and NC. **D-E,** Rescue assays analyzed the expression of SLC1A5 in K-1 cotransfected with si-ABHD11-AS1and miR-199a-5p inhibitor on mRNA and protein levels. **F,** Correlation between ABHD11-AS1 and SLC1A5.

si-Lnc+miR-in: si-ABHD11-AS1+miR-199a-5p-inhibitor

**Figure S2**

**A,** Relative expression of SLC1A5 in thyroid cancer tissues and paired normal tissues from TCGA database and GSE (66783). **B,** Wound healing assay detected the effect of SLC1A5 knockdown on migration of TPC-1 and K-1 cells (scale bar: 100um). **C-D,** Rescue assays examine SLC1A5 expression in cells cotransfected with si-SLC1A5 and miR-199a-5p inhibitor on mRNA and protein levels. **E-F,** Morphological changes of TPC-1 and K-1 cells after transfection under phase contrast microscopy.

si-mRNA+miR-in: si-SLC1A5+miR-199a-5p-inhibitor
